# Spiking neural networks for real-time mapping of EBV-infected B cells in neuroinflammatory lesions

**DOI:** 10.1097/MS9.0000000000005088

**Published:** 2026-05-06

**Authors:** Sadaf Abdullah, Fareeha Anwar, Mahnoor Fatima

**Affiliations:** a Department of Medicine, Lady Reading Hospital Peshawar, Peshawar, Pakistan; b College of Computer and Information Sciences, Imam Mohammad Ibn Saud Islamic University (IMSIU), Riyadh, Saudi Arabia; c Department of Medicine, Shaheed Ziaur Rahman Medical College, Rajshahi Medical University, Bogura, Bangladesh

*To the Editor,*

The Epstein-Barr virus (EBV) has been identified as the primary infectious agent that causes multiple sclerosis (MS), which stands as the most common neuroinflammatory disorder throughout the world. The study, which examined more than 10 million US military personnel samples, found that individuals testing positive for EBV antibodies faced a 32 times greater chance of developing MS because 801 out of 801 tested cases showed antibody development before they started experiencing symptoms, which proved the virus caused B-cell-related neuroinflammation. B cells infected with EBV enter MS lesions where they induce T cell dysfunction and glial cell activation that results in increased demyelination and neurodegeneration^[^1^]^.

Researchers who study postmortem MS brains through high-dimensional imaging have discovered that EBV markers EBNA1 and LMP1 exist in higher concentrations within neuroinflammatory lesions of progressive MS subtypes. The markers identify B cells while showing distribution to both neurons and glial cells. EBNA1-positive cells maintain close connections to astrocytes with a significant result (*P* = 0.03) while also interacting with microglia, which leads to continuous immune system activation and damage to the blood-brain barrier. Secondary progressive MS lesions show that EBV-positive B cells establish greater connections with M2-like macrophages, which leads to increased chronic inflammation (log_2_ odds ratio: 0.36, *P* = 0.02).

Spiking neural networks (SNNs) function as bio-inspired models that replicate neuronal spike timing to achieve energy-efficient real-time biomedical image segmentation, which helps detect EBV-infected B cells in heterogeneous lesions. The three-stage SNN training scheme, which consists of Artificial Neural Network (ANN) pre-training, SNN conversion, and spike-based fine-tuning, produced better Dice coefficients for brain MRI segmentation tasks that included hippocampal delineation because it reached convergence within fewer time steps compared to traditional ANNs. This system solves the problem that artificial neural networks face in low-power edge devices, which process intraoperative pathology data^[^2^]^.

Recent SNN advancements with spike-based attention mechanisms improve medical image segmentation accuracy by 4.2% in Dice score while maintaining <50 ms inference latency on GPUs, which proves optimal for real-time histopathology of neuroinflammatory lesions. The models embedded into DenseNet backbones assist in detecting sparse EBV markers within B cells through their ability to monitor time-based biological changes while exceeding baseline performance during low-contrast testing. The technology allows for identification of EBV-related medical conditions during surgical procedures, which then supports precise antiviral treatment recommendations^[^1^]^.

The development of neural networks, which enable real-time segmentation of EBV-infected B cells, will revolutionize the detection of neuroinflammatory lesions found in MS brain tissue, thus delivering significant economic advantages to the field of neurology. The research of Lanz *et al*^[^3^]^ discovered that EBV-infected B cells drive MS neuroinflammation through their presentation of EBNA1 antigens, which enables U-Net-based convolutional neural networks to detect B cells automatically during live imaging and surgical biopsy procedures. The system switches from expensive manual histopathology work, which costs between $500 and $1000 for each sample, to Artificial Intelligence (AI)-powered analysis that costs under $50 while it completes diagnoses within seconds instead of days, which decreases neurologist tasks by 70%. The deployment of scalable cloud services allows people in areas with limited resources to access treatment, which helps prevent MS progression that costs $85 000 each year per patient through early treatment that eventually generates billions in worldwide healthcare savings^[^3^]^.

Neural networks can potentially perform real-time segmentation tasks for EBV-infected B cells in neuroinflammatory lesions, yet researchers question their ability to adapt to the changing patterns of central nervous system tissues, which MS studies have shown. Läderach *et al*^[4]^ demonstrate that EBV infection drives T-bet+ CXCR3+ B cells to infiltrate the CNS, forming perivascular aggregates that trigger T-cell-mediated neuroinflammation, yet their histological analyses relied on static immunohistochemistry rather than live imaging. The use of neural networks for real-time operations requires comprehensive instruction using multiple datasets that track lesion changes throughout acute flare periods, but this specific data does not exist in current neurostudies, which results in overfitting problems because researchers use fixed samples and postmortem data. The clinical utility of this gap suffers because MS patients experience false positives and missed detections when their lesion microenvironments fluctuate due to blood-brain barrier breaches. Future models must integrate multimodal, real-time CNS imaging to bridge this translational divide^[^4,5^]^.

We propose that SNN–based diagnostic tools be incorporated into multicenter studies supported by dedicated funding for the development of neuromorphic hardware. Collaborative efforts between neuroimmunologists and AI specialists should prioritize the creation of high-quality EBV-infected B-cell segmentation datasets, such as those curated by the National Library of Medicine. Given the rising global burden of MS, regulatory and policy-level support is essential to advance SNN-based pathological assessment methods, which may ultimately contribute to improved disease monitoring and control of disease progression.

## Data Availability

Not applicable to this study.
